# Vitamin D for the prevention of diseases in children: A rebuttal to the 2024 Endocrine Society Clinical Practice Guideline

**DOI:** 10.3389/fendo.2025.1578609

**Published:** 2025-05-29

**Authors:** Benjamin Udoka Nwosu

**Affiliations:** Division of Endocrinology, Department of Pediatrics, Cohen Children’s Medical Center, Queens, NY, Northwell Health, and the Donald and Barbara Zucker School of Medicine at Hofstra/Northwell, Hempstead, NY, United States

**Keywords:** vitamin D, children, guidelines, dark-skinned people, pregnant & lactating women, newborn

## Abstract

The recent 2024 Endocrine Society Clinical Practice Guideline on Vitamin D for the prevention of diseases has become a source of controversy among medical professionals and the lay public. This Review rebuts the recommendations from this Guideline for infants, children, adolescents, pregnant women, and dark-skinned individuals. It rejects the one-size-fits-all recommendations and provides the data for precision-medicine-guided vitamin D screening and supplementation in these populations.

## Introduction

Recommendations on vitamin D screening and supplementation are of global importance because more than half of the world’s population suffer from vitamin D deficiency, as defined by serum 25-hydroxyvitamin D level of <20 ng/mL, especially during the winter months (1). The publication of the 2024 Endocrine Society Clinical Practice Guideline on Vitamin D for the Prevention of Disease in Children and Adults (2) has raised more questions than provided answers (3). While the recommendations for young children, adolescents, dark-skinned-, and obese individuals were controversial, the lack of recommendations for preterm and term infants is baffling. In the section on children, the Panel concluded that vitamin D supplementation could be used only to prevent rickets and upper respiratory tract infections (2). The Panel tangentially noted ‘a potential role for vitamin D in additional health outcomes affecting children, including autoimmune disease, atopy, and diabetes,’ but failed to explore these areas in greater detail as they did for adult patients. The Panel concluded that they ‘found no randomized controlled trial (RCT) data on the effect of treating this population, i.e., children, with vitamin D to lower the risk of prediabetes and type 2 diabetes (2),’ but were silent on the impact of vitamin D supplementation in type 1 diabetes (T1D) where important work exists (4).

The Guideline was tilted heavily toward adults, with minimal emphasis on pediatric health (2). For example, there was no recommendation for infants, i.e., children <1 year of age, despite their increased height velocity and the need for vitamin D supplementation to optimally mineralize the growing skeleton with calcium and phosphorus. The lack of detailed attention to the crucial role of vitamin D in pediatric health led the Panel to exclude important publications on vitamin D in infants, children, and adolescents (4–11). This, in turn, may have led the Panel to incorrect conclusions on the impact of vitamin D in this population. The extrapolation of results from adult studies that lacked a genuine vitamin D-deficient placebo arm (12) is a disservice to children given their unique needs for vitamin D for both skeletal and extra-skeletal functions.

This Review analyzes the most up-to-date and pertinent vitamin D studies focused on feto-maternal physiology, early childhood, and adolescence. This Review further examines the Panel’s recommendations for dark-skinned adults and their consequences for dark-skinned children who are vitamin D deficient.

## Feto-maternal vitamin D physiology and the 2024 vitamin D guidelines

The Panel made no specific recommendations for fetuses, preterm-, and term infants (2). The Panel’s recommendations on vitamin D during pregnancy focused mainly on maternal needs, not the feto-maternal placental unit. Recommendation #9 states that ‘During pregnancy, we (the Panel) suggest against routine 25(OH)D testing’. In Recommendation #8, the Panel recommended ‘empiric vitamin D supplementation during pregnancy, given its potential to lower the risk of pre-eclampsia, intra-uterine mortality, preterm birth, small for gestational age birth, and neonatal mortality’.

To demonstrate the disconnect between recommending not to check 25(OH)D levels in pregnancy, and the recommendation to use empiric vitamin D supplementation with no endpoint monitoring, I shall review recent studies that demonstrate the importance of monitoring serum 25(OH)D concentrations during vitamin D supplementation in pregnancy and post-partum (neonatal) life.

These studies are predicated on the scientific premise that neonatal vitamin D stores in preterm and term infants depend on maternal vitamin D stores as serum 25(OH)D crosses the placental membranes (9, 10). Thus, neonatal serum 25(OH)D concentrations are 50-70% of maternal vitamin D concentrations (13, 14). Additionally, preterm infants generally have low serum 25(OH)D concentrations from decreased transplacental vitamin D transfer from a deficient mother. Finally, preterm infants are at risk for adverse effects of vitamin D deficiency as 80% of placental transfer of calcium and phosphorus occurs between 24 and 40 weeks of gestation (15). Thus, enhanced, not empiric, vitamin D supplementation is crucial for bone health in term- and preterm infants.

In the first study, a randomized clinical trial (RCT), Bhalla et al (10) recruited preterm infants born at 27–36 weeks of gestation within 7 days of birth. The preterm infants were then started on vitamin D supplementation given as 400 international units (IU) plus 150–300 IU/kg in breast milk fortifiers if exclusively breastfed, or 190 IU/kg in milk formulas. They were then randomized to either a monitored vitamin D supplementation group with an option to supplement with vitamin D based on serum 25(OH)D concentration or a standard therapy arm. All infants were followed to 40 weeks of post-conception age. The investigators found that a monitored vitamin D supplementation protocol significantly increased the serum 25(OH)D concentration and other metabolic bone parameters, and decreased the risk of developing metabolic bone disease in premature infants (10).

The second study, a double-blind controlled trial of vitamin D supplementation in preterm infants by Tergestian et al (16), compared the efficacy of different doses of vitamin D, 1000 IU versus 400 IU, to raise serum 25(OH)D concentrations. They found that at 40 weeks post-menstrual age, serum 25(OH)D concentration was significantly higher in the 1000 IU arm compared to the 400 IU arm, 47 ng/mL versus 17 ng/mL, p <0.001. Therefore, these 2 RCTs showed that preterm infants receiving 400 IU of vitamin D daily were deficient in vitamin D at 40 weeks post-conceptual age. They also found that 25(OH)D monitoring was central to ensuring adequate vitamin D supplementation and stores in infants, contrary to the Panel’s recommendations (2, 16).

The third study, a randomized controlled trial (9) of 1300 pregnant women who received a placebo or vitamin D at doses of 4200 IU/week, 16–800 IU/week, 28–000 IU/week from the second trimester to delivery and continued vitamin D supplementation until 6 months postpartum; or vitamin D 28–000 IU/week prenatally and until 6 months postpartum. The results showed that rickets occurred in 4.9% of all of the infants; and that the risk for rickets was highest in the placebo group and lowest in the group where the mothers received the highest dose of vitamin D prenatally and post-natally. The investigators concluded that maternal vitamin D supplementation at 28,000 IU per week during the third trimester of pregnancy until 6 months postpartum reduced the risk for infantile biochemical rickets. Given the findings from the above three RCTs, it is concerning that the Panel was unclear on 25(OH)D monitoring and the recommended dose of vitamin D to prevent infantile rickets in their 2024 Guideline (2).

## Vitamin D guidelines and dark-skinned individuals

Another vague area in the Guideline was the Panel’s recommendation for vitamin D supplementation in dark-skinned children, where the Panel was silent, but expected medical practitioners to extrapolate the Panel’s recommendations for adults to children. The Guideline recommendation #13 reads as follows: ‘In adults with dark complexion, we suggest against routine screening for 25(OH)D levels.’ This recommendation is the opposite of the 2011 Guideline that recommended vitamin D screening and treatment in dark-skinned individuals. It is disturbing that the 2024 Panel recommended discontinuing vitamin D screening in dark-skinned individuals because the Panel ‘found no randomized clinical trials that addressed the question of screening with 25(OH)D in adults with dark skin complexion (17).’ Interestingly, this was the opposite scenario in the light-complexioned population where the Panel identified RCTs to justify discontinuing 25(OH)D screening for vitamin D status. It appears illogical for the Panel to justify their decision in the white population with the availability of justifying data; and then justify the same decision in the dark-skinned population with lack of justifying data. This is surprising given the knowledge that dark-skinned individuals have significantly lower serum 25(OH)D levels compared to their white counterparts (17, 18). It is also surprising that no RCT was conducted on the utility of screening for vitamin D status with 25(OH)D in dark-skinned individuals from 2011 to 2024, a period of more than two decades, while several RCTs were conducted in the white population.

To examine the potential impact of the Panel’s recommendation not to screen for vitamin D status with 25(OH)D in dark-skinned individuals, I shall focus on studies that quantified the impact of vitamin D deficiency on the lives of healthy and wealthy dark-skinned individuals compared to their white counterparts. In the first study, Maroon et al (19) investigated the vitamin D status of 80 elite National Football League (NFL) athletes (84% African American) and the impact of vitamin D status on their health and lifestyle. Though this observational cross-sectional study could not draw causal inferences, it reported a significantly higher rate of vitamin D deficiency in African American NFL players compared to their White counterparts. Secondly, the professional players with vitamin D deficiency were found to be at greater risk for fractures. Finally, players with higher serum 25(OH)D concentrations were more likely to obtain a contract in the NFL. The finding of a correlation of low 25(OH)D levels with increased fracture risk was supported by studies in children, adolescents (20, 21), and adults (11). A cross-sectional study (20) of 18 elite female gymnasts of age 10–17 years found that 83% of those who focused on indoor training had lower vitamin D concentrations and a higher incidence of stress injuries in the year before the testing when compared to their peers with adequate vitamin D levels. Ward et al (21) investigated the relationship between vitamin D status and muscle power and force in 99 girls of age 12–14 years. They reported a positive relationship between serum vitamin D concentrations and muscle power and force. Close et al (11) conducted a randomized placebo-controlled trial with 61 professional male athletes and 30 age-matched non-athletes. They reported that 62% of the athletes were vitamin D deficient at baseline. Following vitamin D supplementation, their serum 25(OH)D rose from 11.6 ± 10 to 41.2 ± 10 ng/mL, P <.01. This increased vitamin D level was associated with increased performance levels for sprints and vertical jumps compared to their baseline level, and to the placebo group. These studies suggest that vitamin D deficiency, as measured by 25(OH)D levels, has a measurable impact on performance, the likelihood of sustaining injuries, and the prospect of building a successful career as an athlete. These conclusions could likely be extrapolated to the general population.

## Vitamin D guidelines and diabetes in children and adolescents

Another deficient section of the 2024 Guideline is on the recommendations for diseases such as diabetes and autoimmune conditions in children and adolescents. The Panel (2) stated that they ‘found no randomized controlled trial (RCT) data on the effect of treating this population, i.e., children, with vitamin D to lower the risk of prediabetes and type 2 diabetes,’ but were silent on type 1 diabetes (T1D) where important studies have been done recently (4).

**Figure 1:**
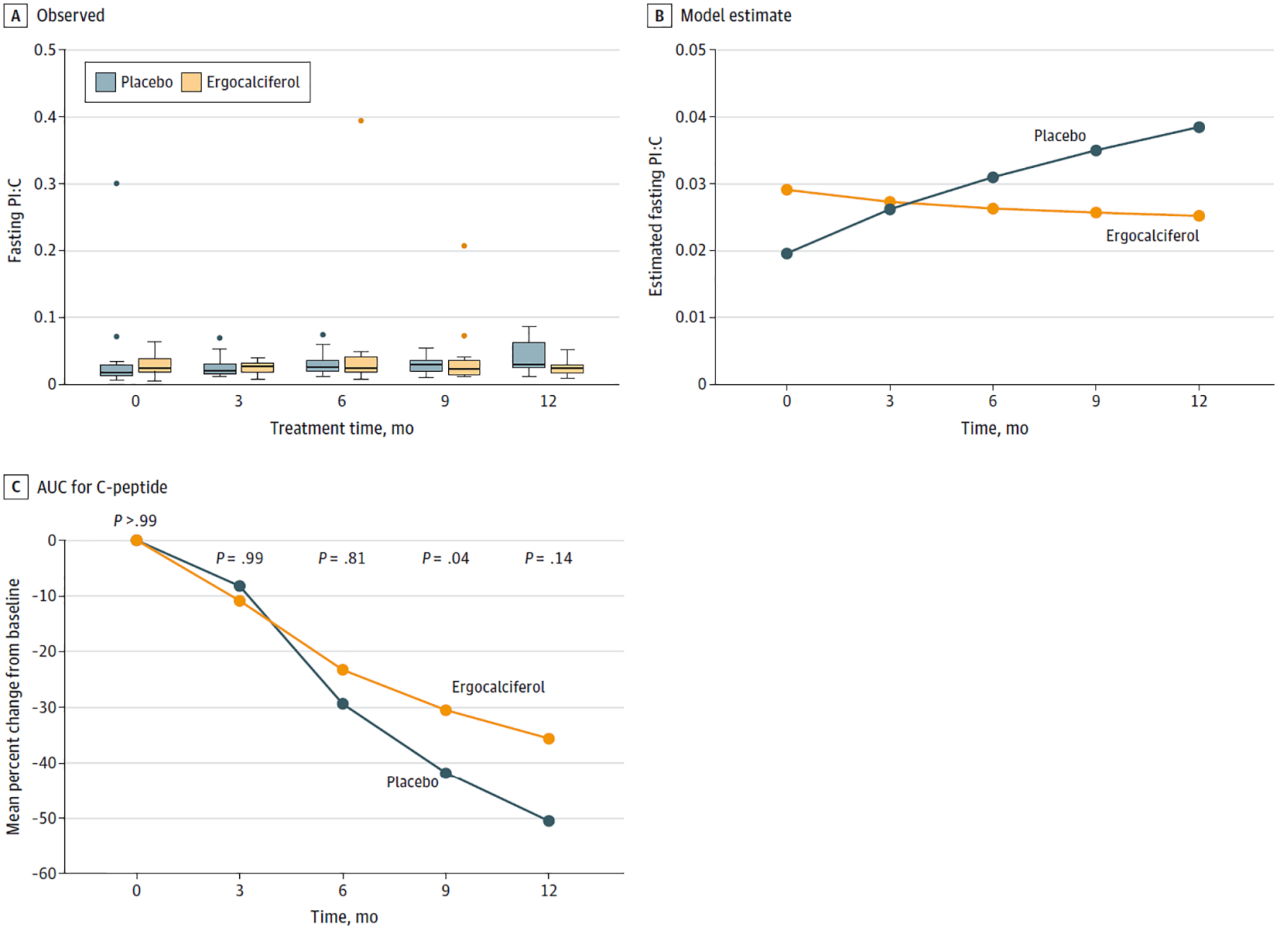
**(A)** Box plots and **(B)** graphs of the fasting proinsulin to C-peptide (PI:C) ratios, and **(C)** a graph comparing the percentage decline from baseline in the area under the curve (AUC) for C-peptide between the placebo and ergocalciferol arms. **(A)** depicts the observed values of the PI:C ratios, and **(B)** shows the model-predicted values. We generated the trends from a repeated-measure generalized linear model of fasting PI:C ratios. The number of repeated-measure observations was 149 from 36 subjects (18 per group). There were 3 observed values greater than 0.2, which were considered extreme outliers and were removed. See outliers in **(A)**. The remaining observations ranged from 0.0053 to 0.0869. The error distribution was normal, the repeated measure correlation was unstructured, and the link function was logarithmic, with the difference in trends between the 2 groups set at a significant *p* value of 0.01. **(C)**, based on the overall analysis of the trends, showed that ergocalciferol significantly slowed the decline in percentage AUC C-peptide from baseline compared to placebo, p=0.03.

For example, in a 12-month RCT of vitamin D to prolong residual β-cell function in children and adolescents, ages 10–21 years, with new onset T1D, Nwosu et al. reported that vitamin D supplementation could prevent the long-term complications of T1D by prolonging the honeymoon phase of T1D(7). The participants received either high dose ergocalciferol, 50,000 IU per week for 2 months, and then biweekly for 10 months, or placebo. In this RCT with a genuine vitamin D deficient placebo group (12) who received no accessory vitamin D supplementation, ergocalciferol significantly decreased fasting proinsulin to C-peptide (PI:C) ratio versus placebo (mean [SE], −0.0009 [0.0008] vs 0.0011 [0.0003]; *P* = 0.01 for the monthly overall difference in trends (**Figure 1A**). Ergocalciferol also significantly decreased the percent change from baseline in the area under the curve (%ΔAUC) of C-peptide compared to placebo, (−28.4 [6.2]; *P* < .001 vs −41.5 [5.9]; *P* < .001), with a significant reduction in monthly overall temporal trends (mean [SE], −2.8% [0.7] vs −4.7% [0.6]; *P* = 0.03 (**Figures 1B, C**). Nwosu et al. had previously reported (22) that vitamin D, ergocalciferol, significantly decreased tumor necrosis factor-alpha (TNF-α) concentrations, the temporal trends in both A1c and the insulin-dose adjusted A1c (IDAA1c) levels, a marker of residual β-cell function. These results showed that vitamin D protects the β-cells and reduces A1c and IDAA1c trends, and thus could possibly prevent long-term complications of T1D.

Unfortunately, this and other important pediatric studies in the field were excluded from the 2024 Guideline. The narrow definition of ‘disease prevention’ by the Panel ignores the disease-modifying properties of vitamin D, which, as shown in the above study, protect the β-cells and helps prevent the severity of long-term complications of T1D in children and adolescents.

The Panel’s focus on rickets and URI as the only two diseases amenable to vitamin D supplementation in children is a missed opportunity to educate health care professionals and the public on recent works on the salutary effects of vitamin D on diseases of children, such as T1D. The Guidelines may not only empower insurance companies to decline screening tests for vitamin D deficiency, but could also discourage important research on the impact of vitamin D on diseases of children and adolescents. The Panel’s sole focus on large RCTs for its recommendations is misleading as these large RCTs have significant limitations that include a lack of a true vitamin D-deficient placebo arm (12) which most likely led to their inconclusive results on the impact of vitamin D on human diseases. Additionally, large RCTs are easier to conduct in the adult population than in children, Therefore, smaller, well-conducted RCTs in children should be given similar consideration as the large RCTs in adults.

In conclusion, the 2024 Guideline on vitamin D screening and supplementation neglected the comprehensive needs of infants, children, and adolescents rigorously as they did for the adult population. Such deficiencies in a widely circulated Guideline could jeopardize access to medical care, forestall research on diseases of children, and lead to poor health outcomes from insufficiently treated, prolonged vitamin D deficiency in children and adolescents.
